# Dynamic inferior olive activation in a cognitive task: an fMRI study

**DOI:** 10.1007/s00429-025-02933-5

**Published:** 2025-05-21

**Authors:** Annakarina Mundorf, Laura C. Rice, Jutta Peterburs, John E. Desmond

**Affiliations:** 1 Department of Neurology, Division of Cognitive Neuroscience, Johns Hopkins University School of Medicine, Baltimore, MD USA; 2 Institute for Systems Medicine and Department of Human Medicine, MSH Medical School Am Kaiserkai 1, 20457 Hamburg, Germany; 3 Center for Neurodevelopmental and Imaging Research, Kennedy Krieger Institute, Baltimore, MD USA

**Keywords:** Error processing, Prediction, Cognition, Teaching signals, Brainstem

## Abstract

The inferior olive provides powerful inputs to the cerebellum hypothesized to support cerebellar learning and error detection. Given cerebellar involvement in verbal working memory and the close interplay with the inferior olive, the inferior olive is likely also involved in verbal working memory. In order to elucidate the inferior olive’s role in verbal working memory, we utilized an MRI-based Sternberg verbal working memory task which involved learning novel vs repeated sequences. As hypothesized, inferior olive activation was stronger during encoding and retrieval compared to maintenance, especially for novel compared to repeated sequences, indicative of diminished inferior olive activity with stimulus repetition. Results also revealed differential inferior olive activation during retrieval, with increased activation on matching probes for novel and on non-matching probes for repeated sequences. This underlines the crucial role of the inferior olive in novel information encoding and error feedback, and that conditions triggering strong inferior olive responses can change as a function of novelty.

## Introduction

The inferior olive provides one of the most powerful synapses of the brain to the cerebellum via its Purkinje cell-targeted climbing fibers. These fibers enter the cerebellum through the contralateral inferior cerebellar peduncle (Goodlett and Mittleman 2017), and the effects of their synapses on Purkinje cells can be readily observed electrophysiologically in the form of complex spikes. The convergence of granule cell parallel fiber and climbing fiber inputs on Purkinje cells has played a prominent role in theoretical models of cerebellar function such as those by Marr and Albus (Marr 1969; Albus 1971). In such models, co-occurrence of parallel fiber inputs with climbing fiber teaching/error inputs has been hypothesized to modify the efficacy of parallel fiber/Purkinje cell synapses, thereby providing a hypothetical learning mechanism that has received support from experimental studies (Ito 1982, 1986).

Albus adopted classical conditioning terminology in portraying inferior olive function, describing the climbing fiber burst as an Unconditioned Stimulus (US). Thus, the discovery, about a decade later, that the cerebellum is essential for a fundamental form of associative learning, the classically conditioned eyeblink response (McCormick and Thompson 1984), triggered immediate interest in testing whether the inferior olive provided the climbing fiber US to the cerebellum that was envisioned by Albus (1971) as the teaching signal supporting learning of an association between two stimuli, a conditioned stimulus (CS) and the US. These investigations revealed that inferior olivary neurons fire in response to the normal air puff US (Sears and Steinmetz 1991), that lesions of the inferior olive produced extinction of conditioned responses (CRs) without affecting the eyeblink unconditioned response (UR) (McCormick et al. 1985), and that pairing a tone conditioned stimulus (CS) with stimulation of inferior olive, in the absence of an air puff, was sufficient to support the development of normal eyeblink CRs in a naïve animal (Mauk et al. 1986). These studies thus confirmed the critical role of the inferior olive in forming and maintaining stimulus associations. Subsequent models such as the forward model (Wolpert and Miall 1996; Wolpert et al. 1998; Miall and Reckess 2002; Kawato et al. 2003; Ramnani 2006; Miall et al. 2007; Popa and Ebner 2019), emphasize a role of the inferior olive—which additionally demonstrates high connectivity with motor function-related areas, such as the primary motor cortex, the cerebral peduncle of the midbrain, the pontine basis, the red nucleus, and the posterior limb of the internal capsule (Jang et al. 2012)—in comparing predicted and actual events, thereby allowing it to generate an error signal when a discrepancy exists. Such a role has been supported by electrophysiological evidence in animals demonstrating that introduction of an obstacle during the execution of a routine motor trajectory results in robust inferior olivary responses that signify the error detection from the unexpected event (Gellman et al. 1985).

These two processes ascribed to inferior olive function, i.e., promoting stimulus associations and error detection/correction, can be found in certain forms of verbal working memory. For example, the Sternberg task, which is commonly used to investigate verbal working memory, consists of three distinct phases: In the encoding phase, a sequence of letters or numbers is briefly presented. In the subsequent maintenance phase, the subject must internally keep the verbal stimuli in mind for a period of time typically by a covert or overt articulatory loop. In the retrieval phase, a probe stimulus is presented and the subject must decide if the probe is a match or a non-match to one of the items kept in mind.

Given evidence from both neuroimaging and patient studies of cerebellar involvement in verbal working memory (Kirschen et al. 2008; Peterburs et al. 2010; Stoodley 2012; Tomlinson et al. 2014), it is reasonable to hypothesize that the inferior olive would respond to this task due to its powerful inputs to the cerebellum and the close interplay between the inferior olive and the cerebellum (Goodlett and Mittleman 2017). Furthermore, we hypothesize that the phase-dependent responses of the inferior olive can be predicted based on the task phase requirements and the proposed functions of the inferior olive. That is, in the Sternberg task, the encoding phase emphasizes the rapid learning of a sequence of items and would therefore benefit from facilitation of stimulus associations that the inferior olive could provide. Previous studies from our lab (Chen and Desmond 2005) and others (Chein and Fiez 2001; Durisko and Fiez 2010) have found that the superior cerebellum activates robustly during the encoding phase, potentially reflecting the formation of stimulus associations that would benefit sequencing and rapid subsequent rehearsal.

The maintenance phase is characterized by covert articulatory rehearsal, and our previous studies have found inferior cerebellar activation during this phase (Chen and Desmond 2005; Peterburs et al. 2019). In prior work we hypothesized that the maintenance phase might involve error correction, based on the discrepancy between the desired and actual rehearsal trajectories (Desmond et al. 1997) in which case inferior olivary responses might be observed. However, more recent work has emphasized that the phonological loop rehearsal process during this phase is likely benefitting from the cerebellum’s hypothesized feedforward predictions of the articulatory sequence, similar to how rapidly coordinated limb movement is thought to benefit from such predictive control. In support of this prediction hypothesis, we found that cerebellar, but not occipital, transcranial magnetic stimulation administered at specific points during a guided rehearsal procedure significantly impaired a subject’s ability to recognize the correct letter in a sequence of letters (Sheu et al. 2019). If cerebellar activation observed during the maintenance phase is predominantly reflecting this feedforward prediction rather than error correction, then, by forward model accounts (Wolpert et al. 1998; Ramnani 2006), inferior olivary responses would be less prominent in this phase.

The retrieval phase involves making a decision as to whether the probe matches a letter being held in mind. In the cerebellum, the retrieval phase for the Sternberg task as described here tends to show less activation than that seen in the encoding or maintenance phases, unless additional executive demand is imposed (Chen and Desmond 2005; Marvel and Desmond 2010). However, from the perspective of hypothesized inferior olive involvement in error detection, the retrieval phase requires evaluation of the expectation that the probe matches one of the items being held in mind. Trials in which the probe does not match would therefore constitute an error condition, in which case we would expect robust inferior olive activation in the retrieval phase to non-matching probes. Given this evaluation of task requirements and presumed inferior olivary function, we hypothesize a task phase dependent activation of the inferior olive, with significant inferior olivary activation during the encoding phase of the task, and during the retrieval phase on non-match trials, relative to the maintenance phase of the task. We further hypothesize that inferior olive activation will diminish with repetition of the stimuli, and thus, if a repeating sequence of items is presented on some trials, those trials would exhibit reduced inferior olivary activation during encoding and retrieval phases relative to trials in which completely novel sequences were presented. Based on previous findings that link predominantly right cerebellar cortex to verbal working memory (Desmond et al. 1997; Kirschen et al. 2008; Stoodley 2012; Tomlinson et al. 2014; Peterburs et al. 2019), we will focus on the left inferior olive as its axons cross over to the right cerebellar cortex (Bourrat and Sotelo 1988; Marillat et al. 2004). To this end, healthy participants performed a Sternberg Verbal Working Memory Task, while inferior olive activation was measured.

## Materials and methods

### Subjects

Twenty healthy adult volunteers were recruited from the Baltimore community, and nineteen were included in the analysis (12 females, 7 males; mean age 25.1 ± 2.9 years, age range 19–30 years; mean educational attainment: 17.1 ± 1.6 years, range: 14–20 years). One participant had to be excluded due to low performance (< 60% accuracy averaged across all three task runs). Participants were native English speakers, right-handed according to self-report, and had normal or corrected-to-normal vision. Furthermore, all subjects had no history of neurological or psychiatric illness or head trauma and were not currently taking any medication affecting the central nervous system. For MRI scanning, additional exclusion criteria were applied: (self-reported) claustrophobia, implanted electric or ferromagnetic devices, and pregnancy. Before participation, subjects gave written informed consent. Afterward, they were compensated for their participation and travel expenses. The study was approved by the Johns Hopkins University School of Medicine Institutional Review Board and is in line with the Declaration of Helsinki.

### Sternberg verbal working memory task

**Fig. 1:**
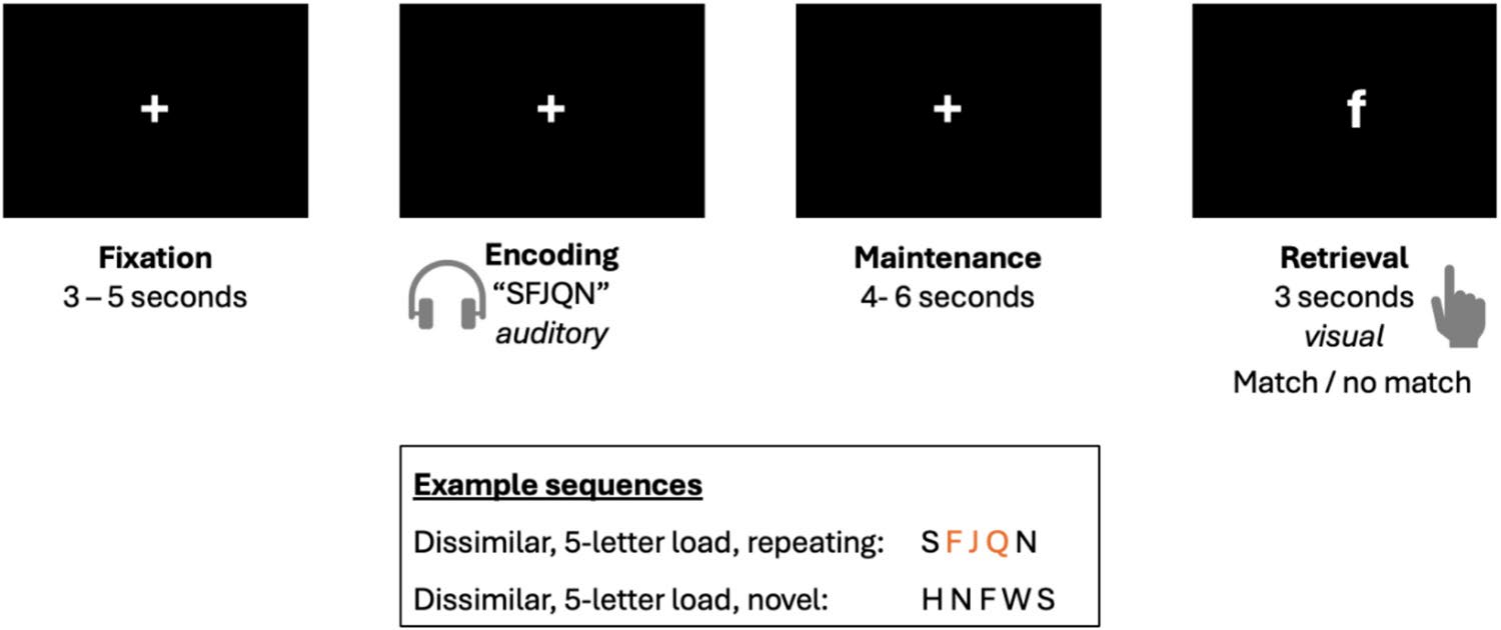
Stimulus presentation and the three phases of working memory: encoding, maintenance, and retrieval. Examples of phonologically dissimilar letters with a 5-letter load and either repeating or novel sequences are shown. Participants had to encode the auditorily presented information (encoding phase), maintain the information over a brief delay interval (maintenance phase), and respond to a visually-presented probe stimulus (retrieval phase)

We re-analyzed the acquired data that was previously published (Peterburs et al. 2019). In the current study, the focus lies on the novel and repeating dissimilar 5-letter load conditions. In these conditions, participants were aurally presented with five phonologically dissimilar letters that were either completely novel sequences or contained a repeating 3-letter sequence that occurred at the beginning, in the middle, or at the end of the sequence. Each trial started with a fixation cross presented for a jittered inter-trial interval of 3–5 s (Fig. 1). Then, five digitally recorded spoken letters pronounced by a male actor, downloaded from a royalty-free website (soundbible.com/2009-A-Z-Vocalized.html), were presented sequentially as encoding stimuli. The letters were chosen from a pool of phonologically dissimilar letters (F–H–J–N–Q–R–S–W) with half of the trials containing a repeating sequence of three letters (F–J–Q), while in the other half, new letters were presented. The auditory stimuli were presented via noise-canceling MR-compatible headphones (OptoActive IITM, Optoacoustics Ltd., Moshav Mazor, Israel) enabling a reduction of the scanner noises to 70–77 dB, with the output stimulus sound set to 85 dB. Next, a blank screen was presented for a jittered interval of 4–6 s, and the subjects were supposed to rehearse the letters (maintenance phase). Lastly, in the following retrieval phase, subjects were presented with a probe letter for 3 s and had to indicate if the letter was a match or non-match of one of the letters presented in the encoding phase by pressing the appropriate response button with either their right index (“yes”) or middle (“no”) finger. Matching and nonmatching probes in the retrieval phase each occurred with a 50% probability. As behavioral parameters, accuracy and reaction time were recorded per trial and participants were instructed to respond as accurately and quickly as possible. The complete task was comprised of three runs with 72 trials each, resulting in a total of 216 trials, from which 54 trials (27 novel, 27 repeating) that were relevant to this report were extracted. Trials for the novel and repeating conditions occurred in a random order. The jittering of the maintenance phase and inter-trial interval has been used in our previous work (Chen and Desmond 2005), and in that study we used simulations to verify that spm could distinguish between hypothetical voxels responding to the encoding, maintenance or retrieval phase (after convolution with an HRF), or some combination of these phases.

### MRI data acquisition

MRI data were acquired as described in our previous study (Peterburs et al. 2019). A 3.0-T Philips Intera scanner (Philips, Eindhoven, NL, USA) was used. The structural MRI protocol consisted of a T1- weighted MPRAGE (TR = 6.97 ms; TE = 3.3 ms; TI = 982 ms; flip = 8°, inplane resolution = 0.75 mm; slice thickness = 1 mm; 170 sagittal slices; FOV = 240 mm; 1 NEX). Functional magnetic resonance imaging (fMRI) data were collected using a T2*- weighted gradient echo EPI pulse sequence (TR = 1000 ms; TE = 30 ms; flip = 61°; inplane resolution = 2.75 mm; slice thickness = 6 mm skip 1 mm; 20 oblique-axial slices; FOV = 240 mm; 1 NEX). fMRI images were acquired as oblique-axial slices rotated 25° clockwise with respect to the anterior commissure-posterior commissure line to adequately acquire brainstem, cerebellum, and neocortex. The number of acquired volumes within each block ranged from 944 to 953. At the beginning of each block, the start of the fMRI scan and the start of the experiment were synchronized with E-prime 2 software (Psychology Software Tools, Inc., Sharpsburg, PA, USA).

### Analysis of functional MRI data

For each subject, standard preprocessing steps were performed in SPM12, implemented in MATLAB (R2017b), including timing correction, motion correction, functional–anatomical coregistration, segmentation-based MNI normalization, and spatial smoothing. Individual statistical maps were computed using the general linear model approach as implemented in SPM12 (Penny et al. 2011), with high-pass filtering of 128s. In this analysis, encoding, maintenance and retrieval phases were modeled for each trial type, and the retrieval phase was divided into trials in which the probe stimulus matched one of the letters in the encoding phase and trials that were a non-match. Of specific interest were the beta values for the encoding, maintenance, retrieval-match, and retrieval-non-match events in the regression for the novel and repeating sequences. The mean beta values for these events were extracted from a left inferior olive MNI-registered region of interest (ROI). This ROI was obtained from the Brainstem Nuclei Atlas from the Brainstem Imaging Laboratory (Bianciardi 2021). The left inferior olive ROI from this dataset was thresholded at 0.35 probability and binarized into 0 or 1 values for extracting BOLD signal values (Bianciardi 2021). The mean beta values extracted from the ROI were then analyzed using conventional statistical software, as described below. Additionally, in SPM12, specific first-level contrasts were created to examine differences between encoding and maintenance, retrieval and maintenance, and the interaction of repetition (novel, repeating) with retrieval type (match, non-match). The resulting contrasts were entered into group-level random effects analyses using one-sample Student’s t-tests against a contrast value of zero at every voxel at the whole-brain level in order to visualize inferior olive activation.

### Statistical analyses

As behavioral performance measures, individual percent accuracy and mean of subject median reaction time were calculated over the entire session. For both measures, repeated measures ANOVA was performed with novelty and retrieval probe match type as within-subject variables. For functional brain activation, the beta values for the regressors described above were used as the dependent variable for a 4 × 2 repeated measures ANOVA, with within-subject factors of task phase (encoding, maintenance, retrieval match, retrieval non-match) and novelty (novel or repeated sequence). We hypothesized a main effect of phase and a phase x novelty interaction. We also tested whether the mean beta values were significantly different from 0 with the goal of determining for each phase if the inferior olive was significantly activated. In a subsequent step, we performed contrast analyses to further characterize phase and sequence novelty dependent patterns of activation, including an interaction between sequence novelty and retrieval probe type. Effect sizes are reported as Cohen’s *d* for t-tests and as partial eta^2^ (*η*_*p*_^*2*^) for ANOVA and characterized as small (*d* ≤ ± 0.5; *η*_*p*_^*2*^ = 0.01), medium (*d* ≤ ± 0.8, *η*_*p*_^*2*^ = 0.06), and large (*d* ≥ ± 0.8; *η*_*p*_^*2*^ = 0.14) (Cohen 1988). All statistical analyses were performed with Statistica 13.

In the current study, we re-analyzed data from a previous study (Peterburs et al. 2019), which included 19 participants and had a power of 80% for detecting the primary effects. This power analysis was based on the sample size of the original study, but it should be noted that the analysis was not specifically optimized for detecting activations in the inferior olive, as this was not a focus of the original work. As such, while the power analysis provides a useful estimate for the current dataset, it is not tailored to the particular regions of interest in this re-analysis.

To further assess the robustness of the findings, a post hoc power analysis using G*Power (Faul et al., 2007) was conducted to evaluate the statistical power of the 4 × 2 repeated measures ANOVA with 19 participants. Based on the observed effect size for the significant task phase main effect (ηp^2^ = 0.37; f = 0.77) and the significant phase × novelty interaction (ηp^2^ = 0.16, f = 0.44), the analysis indicated effectively 100% power to detect significant effects (1.0 and 0.996, respectively), confirming the robustness of the results and suggesting that the sample size was adequate to detect the observed effects. While the sample size of 19 participants in the original study is larger than that used in many previous studies focusing specifically on the inferior olive (Liu et al. 2008; Teki et al. 2011; Wu et al. 2011; Xu et al. 2006), we acknowledge that it may still represent a limitation when interpreting the present results. Future studies with larger sample sizes or more targeted power analyses may help better address the nuances of inferior olive activations.

## Results behavioral performance

### Accuracy

**Fig. 2:**
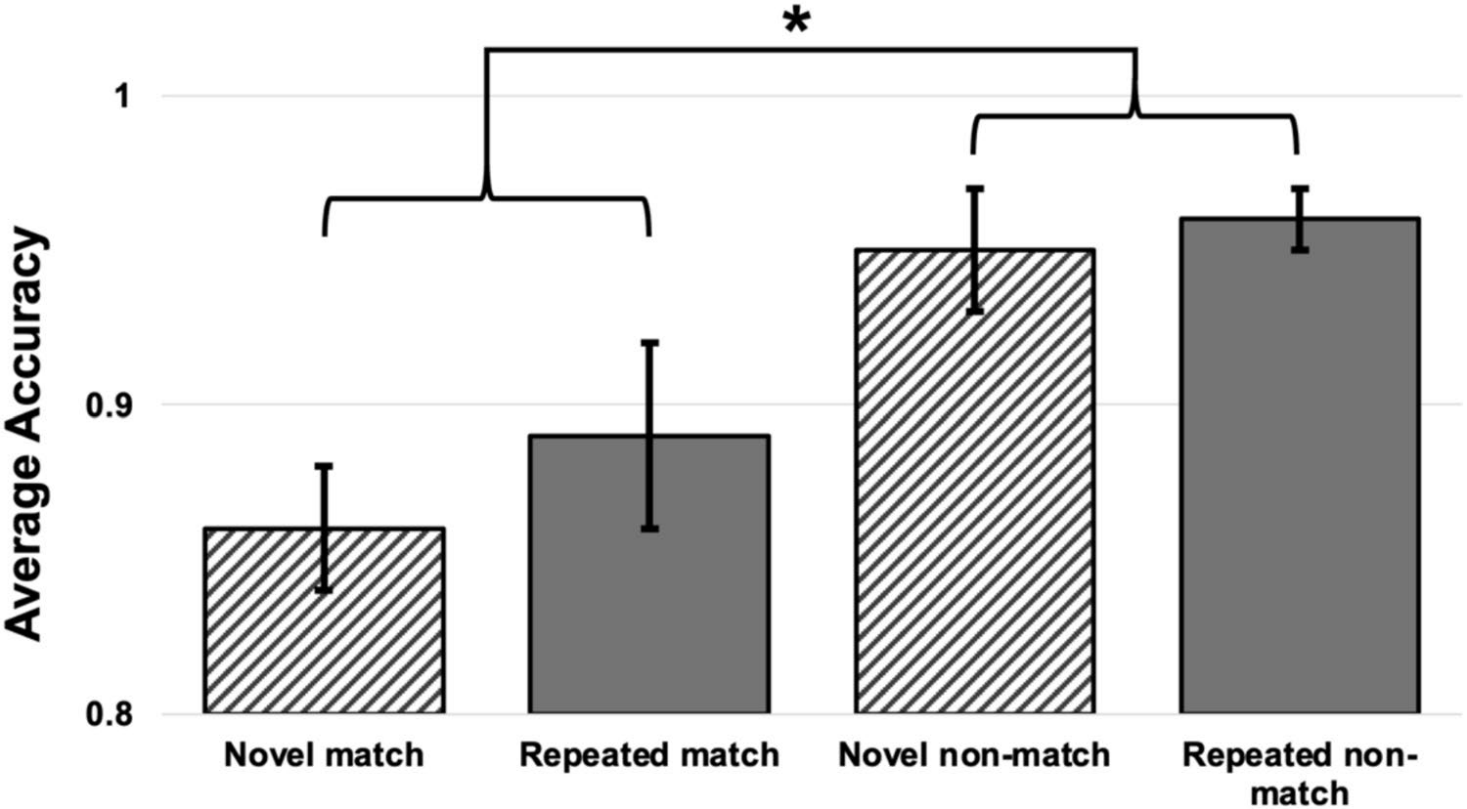
Averaged accuracy averaged over three runs, separately for matching and non-matching trials. Overall, accuracy was very high (above 85%) in all conditions: novel sequence, match; repeated sequence, match; novel sequence, non-match; repeated sequence, non-match. A significant main effect of probe type emerged, with matching probes having a mean accuracy of 87.9% ± 2.9% SE and non-matching probes having a mean accuracy of 95.4% ± 1.9% SE. Bars represent the standard error. *p = 0.002

Overall, accuracy was very high, above 85%, in all conditions (Fig. 2). A 2 × 2 repeated measures ANOVA with novelty (novel vs repeating) and retrieval probe type (match vs nonmatch) revealed only a main effect of probe type (F(1, 18) = 14.06, *p* 0.002, *η*_*p*_^*2*^ = 0.44), with matching probes having a mean accuracy of 87.9% ± 2.9% Standard Error (SE) and non-matching probes having a mean accuracy of 95.4% ± 1.9% SE. All other effects were non-significant (all p > 0.05).

### Reaction time

**Fig. 3:**
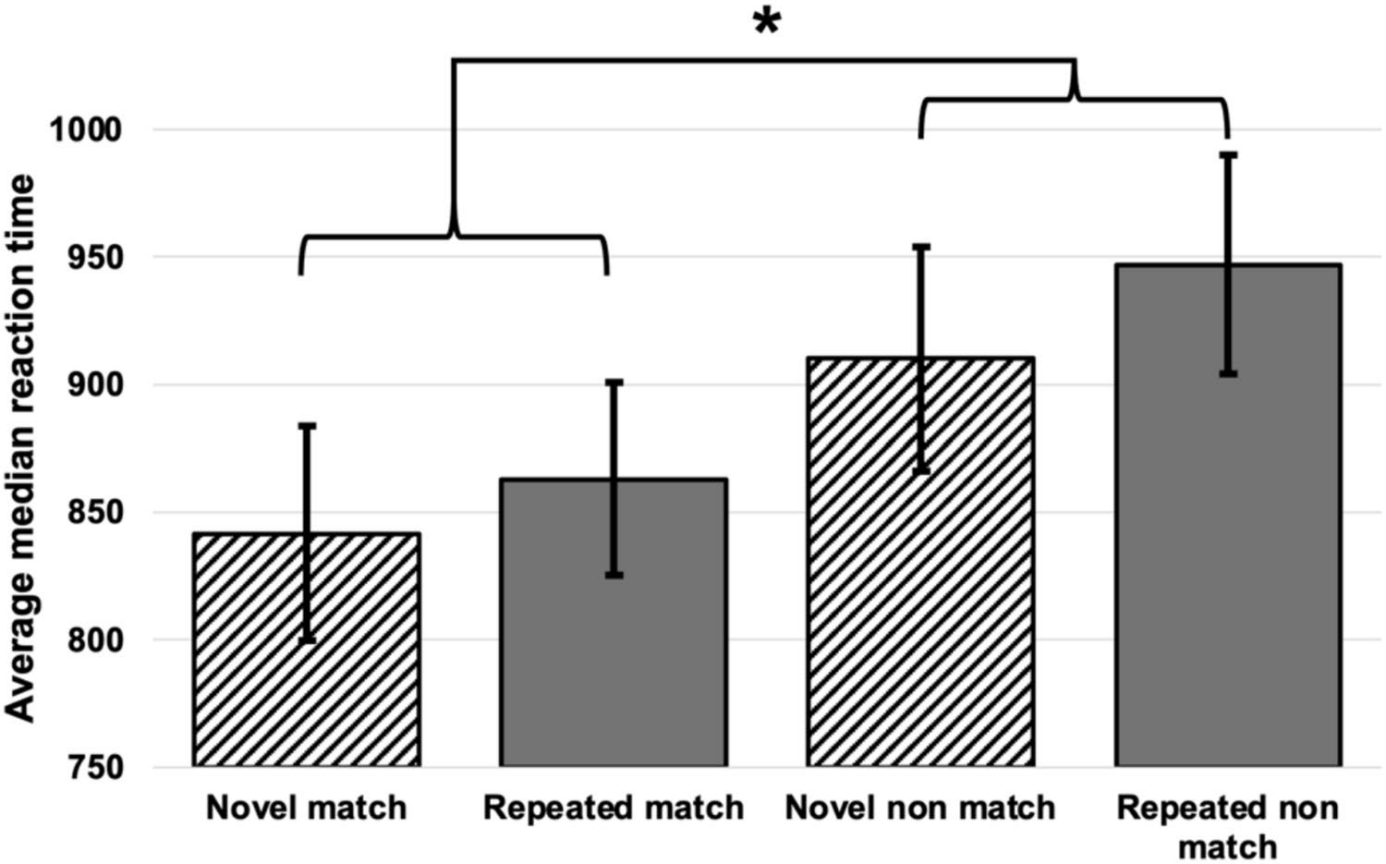
Averaged median reaction time for correct trials in milliseconds averaged over three runs, separately for matching and non-matching trials. Shown are the results of the conditions: novel sequence, match; repeated sequence, match; novel sequence, non-match; repeated sequence, non-match. A significant main effect of probe type was evident, with reaction times for matching probes (852.3 ms ± 52.7 ms SE) significantly lower than those for non-matching probes (928.7 ms ± 59.8 ms SE). Bars represent the standard error. *p = 0.004

Individual median reaction time in milliseconds on correct trials was averaged across the three runs and calculated separately for novel vs repeating trial types, and matching vs non-matching retrieval probes. Results for these trial types are presented in Fig. 3.

A 2 × 2 repeated measures ANOVA with novelty (novel vs repeating) and retrieval probe type (match vs nonmatch) revealed only a significant main effect of probe type (F(1, 18) = 10.70, *p* = 0.004, η_*p*_^2^ = 0.37). Reaction times for matching probes (852.3 ms ± 52.7 ms SE) were significantly lower than those for non-matching probes (928.7 ms ± 59.8 ms SE). All other effects were non-significant (all *p* > 0.05).

### Co-registration of individual anatomical data and the inferior olive ROI of the brainstem nuclei atlas

**Fig. 4:**
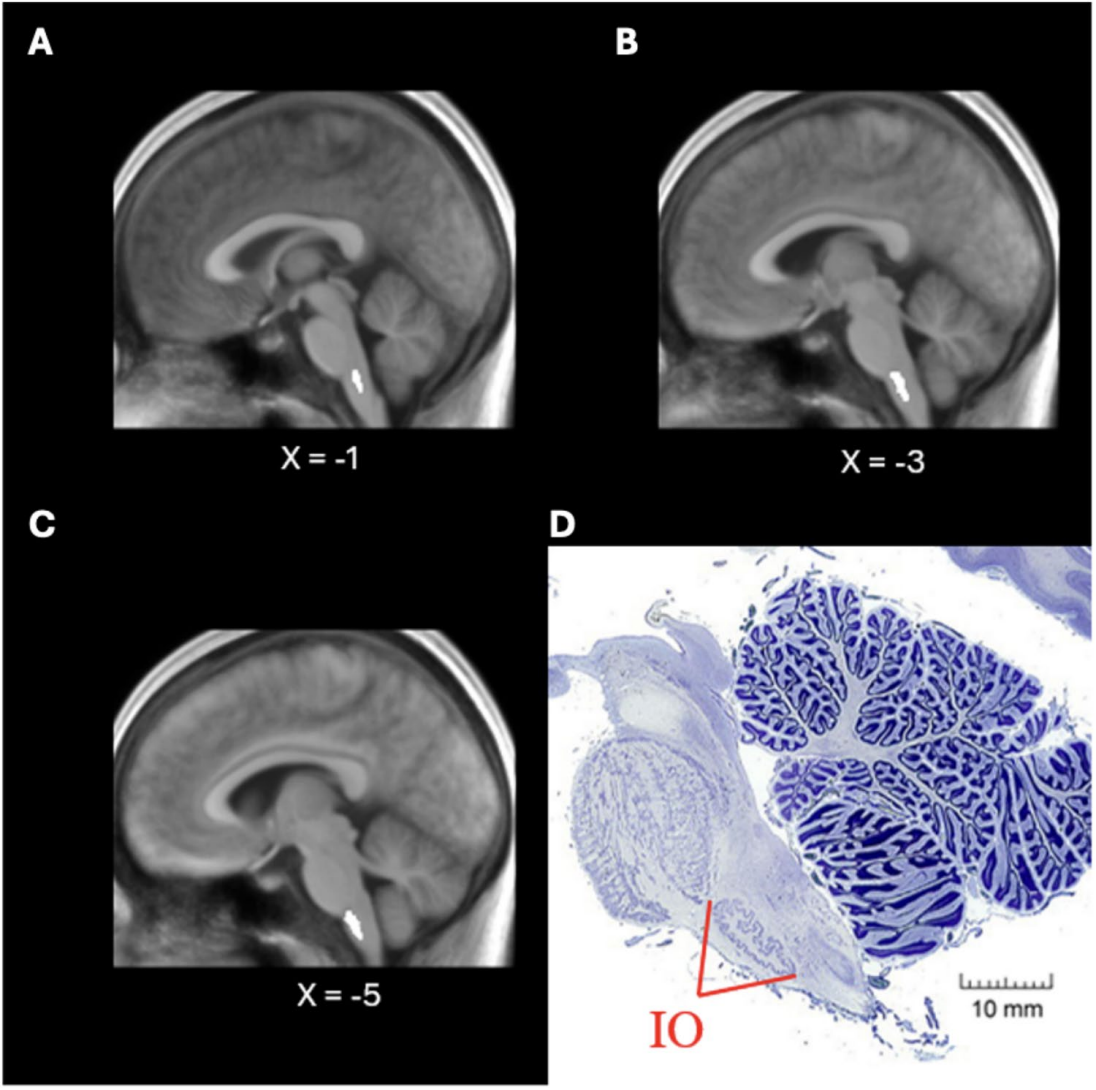
Inferior Olive ROI of the Brainstem Nuclei Atlas. MNI-normalized MPRAGE volume averaged across all subjects and sliced sagitally at the X = – 1, – 3, and – 5-mm coordinate (**A**, **B**, **C**, respectively). The localization of the inferior olive ROI from the Brainstem Nuclei Atlas has been enhanced in these sections by artificially brightening the region. **D** Structural appearance of the inferior olive (IO) seen on histologically stained sections. The location depicted in **A**–**C** corresponds well with the structural appearance in **D** Adapted with permission from https://brainmuseum.org/, Specimens used for this publication are from the Defense Health Agency Neuroanatomical Collections Division of the National Museum of Health and Medicine, the University of Wisconsin and Michigan State Comparative Mammalian Brain Collections supported by the US National Science Foundation

Figure 4A–C shows the MNI-normalized MPRAGE volume averaged across all subjects and sliced sagitally at the X = – 1, – 3, and – 5-mm coordinate (left side). The localization of the inferior olive ROI from the Brainstem Nuclei Atlas has been enhanced in these sections by artificially brightening the region. The location depicted corresponds well with the structural appearance of the inferior olive seen on histologically stained sections (Fig. 4D).

### Phase-dependent inferior olive activation

**Fig. 5:**
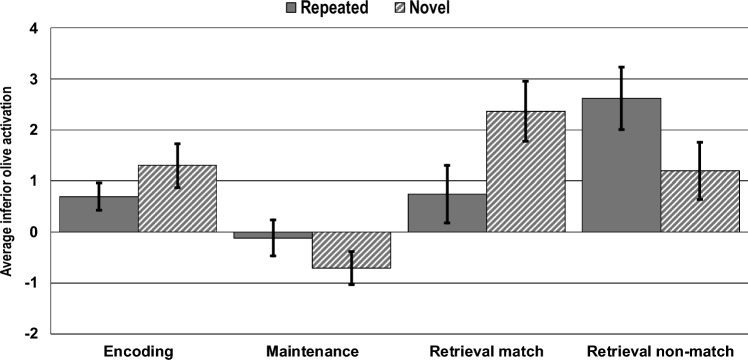
Average inferior olive activation during encoding, maintenance, and retrieval (match and non-match). The average BOLD activation of the left inferior olive is shown during each phase for repeated and novel letter sequences. Activation is evident during encoding for novel sequences (M = 1.30, SE = 0.43) and repeating sequences (M = 0.69, SE = 0.27). Relative to encoding, reduced activation is visible during maintenance for both novel (M = – 0.71, SE = 0.33) and repeating (M = – 0.12, SE = 0.35) sequences. Increased activation was evident during retrieval for novel matching (M = 2.37, SE = 0.59), novel non-matching (M = 1.20, SE = 0.56), repeating non-matching (M = 2.62, SE = 0.62) with lowest activation seen during retrieval for the repeating matching condition (M = 0.73, SE = 0.56). Bars represent the standard error

Repeated measured ANOVA revealed a significant main effect of phase on inferior olive activation (F(3, 54) = 10.51, *p* < 0.001, η_*p*_^2^ = 0.37), no main effect of novelty alone (F(1, 18) = 0.07, *p* = 0.8, η_*p*_^2^ < 0.01), and a significant interaction effect of phase x novelty on inferior olive activation (F(3, 54) = 3.53, *p* = 0.021, η_*p*_^2^ = 0.16). Average left inferior olive activation during encoding, maintenance, and retrieval for novel and repeating sequences is presented in Fig. 5.

Activation is evident during encoding for novel sequences (*M* = 1.30, *SE* = 0.43; one sample *t*-test: *t*(18) = 3.01, *p* = 0.007, *d* = 0.7) and repeating sequences (*M* = 0.69, *SE* = 0.27; *t*(18) = 2.60, *p* = 0.018, *d* = 0.6). The predicted reduction in encoding activation for repeating sequences relative to novel sequences approached significance (F(1, 18) = 3.77, *p* = 0.068, η_*p*_^2^ = 0.17). Relative to encoding, reduced activation is visible during maintenance for both novel (*M* = – 0.71, *SE* = 0.33) and repeating (*M* = – 0.12, *SE* = 0.35) sequences. The encoding-to-maintenance reduction was significant for novel sequences (F(1, 18) = 11.20, *p* = 0.004, η_*p*_^2^ = 0. 38), and approached significance for repeating sequences (F(1,18) = 3.09, *p* = 0.096, η_*p*_^2^ = 0.15). The reduction in activation from encoding to maintenance for novel sequences was significantly greater than that observed for repeating sequences (F(1, 18) = 4.53, *p* = 0.047, η_*p*_^2^ = 0.20).

**Fig. 6:**
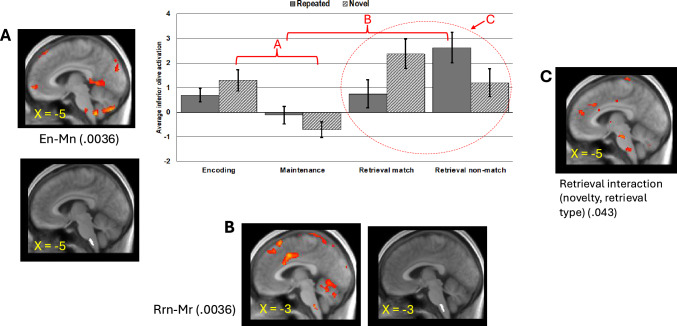
Visual presentation of the three contrast analyses performed for phase and novelty. Shown are examples of average inferior olive activation in the respective phase and the anatomical location of the inferior olive according to the Brainstem Nuclei Atlas from the Brainstem Imaging Laboratory. Red indicates average activation in the individuals. *En* Encoding novel sequences, *Mn* Maintenance novel sequences, *Mr* Maintenance repeated sequences, *Rrn* Retrieval repeated sequences, non-matching probes. The threshold for the activation maps is set to *p* = 0.005 for the retrieval interaction and to *p* = 0.001 for all others

Considering the maintenance phase alone, the activation for repeating sequences was not significantly different from 0 (*t*(18) = – 0.34, *p* = 0.738, *d* = – 0.08), whereas the maintenance phase activation for novel sequences went significantly below 0 (*t*(18) = – 2.19, *p* = 0.042, d = – 0.5). However, direct comparison of novel vs. repeated maintenance phase activation did not reach significance (F(1, 18) = 2.87, *p* = 0.107, η_*p*_^2^ = 0.14); see Fig. 6.

During retrieval, inferior olive activation exhibited an unexpected pattern that appeared to depend on both sequence novelty and retrieval probe type. A post-hoc interaction contrast of sequence novelty (novel vs repeating) and retrieval probe type (match or non-match), with 0 contrast values entered for encoding and maintenance, confirmed that this interaction was significant (F(1, 18) = 4.74, *p* = 0.043, η_*p*_^2^ = 0.21). For the repeating condition, non-match probes during retrieval resulted in significantly greater activation than match probes (F(1, 18) = 4.53, *p* = 0.047, η_*p*_^2^ = 0.20), as originally predicted. For the novel sequences condition, however, the opposite trend, i.e., match > non-match, was present, although a direct contrast of these means was not significant (F(1, 18) = 2.01, *p* = 0.173, η_*p*_^2^ = 0.10). Although retrieval activation for novel matching (*M* = 2.37, *SE* = 0.59; *t*(18) = 4.02, *p* < 0.001, *d* = 0.9), novel non-matching (*M* = 1.20, *SE* = 0.56; *t*(18) = 2.13, *p* = 0.047, *d* = 0.5), and repeating non-matching (*M* = 2.62, *SE* = 0.62; *t*(18) = 4.26, *p* < 0.001, *d* = 0.9) conditions were all significantly greater than 0, the retrieval activation for the repeating matching condition (*M* = 0.74, *SE* = 0.56) was not (*t*(18) = 1.32, *p* = 0.204, *d* = 0.3). For the repeating sequence condition, the retrieval matching activation was not significantly different from the maintenance activation (F(1, 18) = 2.03, *p* = 0.172, η_*p*_^2^ = 0.10) in contrast to the highly significant maintenance to retrieval increase for the non-matching retrieval phase (F(1, 18) = 11.21, *p* = 0.004, η_*p*_^2^ = 0.38). For novel sequences, both matching and non-matching retrieval activations were significantly different from the novel maintenance activations (matching F(1, 18) = 16.38, *p* = 0.001, η_*p*_^2^ = 0.48; non-matching F(1, 18) = 10.99, *p* = 0.004, η_*p*_^2^ = 0.38).

## Discussion

This study examined phase-dependent inferior olive activation in a Sternberg verbal working memory task. Consistent with our hypothesis, results indicate stronger activation during encoding and retrieval compared to maintenance of information. This increase was especially pronounced for novel sequences compared to stimulus repetition, suggesting diminished inferior olive activity with greater learning. Remarkably, results also point towards differential left inferior olive activation during retrieval for matching and non-matching sequences as a function of the novelty of the sequences. Specifically, increased activation was evident for novel sequences in matching trials, whereas activation was increased for repeated sequences in non-match trials. Our original prediction was that repetition would decrease inferior olive activation and that non-match probes would elicit greater activation than match probes. Repetition-related decreases in inferior olive activation were observed during encoding, and for match probes when sequences were novel, but the error-detection-related inferior olive response for non-match trials occurred only after subjects had greater familiarity with the sequence, at which point the inferior olive response to matching probes was not significantly different from 0. The inferior olive therefore exhibited a dynamic pattern of signaling in which a probe that matched one of the encoding letters caused high inferior olive signaling for novel sequences, but once the sequence was familiar, a probe that was different from the sequence elicited the expected inferior olive “error signal”.

Behaviorally, only a main effect of retrieval probe type was found for both accuracy and reaction time, with non-matching probes requiring more time for a response, but responses were more accurate compared to matching probes. In contrast to the novelty x probe type interaction observed in inferior olive activation during the retrieval phase, neither accuracy nor reaction time exhibited such interactions, suggesting that inferior olive activation was not simply mirroring these behavioral variables.

To date, few studies have focused on fMRI responses in the inferior olive. Previous investigations have explored the role of this structure in sequence timing (Xu et al. 2006; Teki et al. 2011), and encoding of temporal information independent of motor behavior (Xu et al. 2006), in accordance with a well-established literature on cerebellar involvement in timing that began with reports of abnormal perception and expression in human cerebellar patients (Ivry et al. 1988; Ivry and Keele 1989). To dissociate the temporal from nontemporal attributes of sensory input, researchers used an event-related fMRI and a perceptual task in which participants were asked to detect changes in timing, spatial orientation, or color of identical visual stimuli presented rhythmically (Liu et al. 2008). The results revealed that the inferior olive activation was specific to a change in stimulus timing, underlining that the inferior olive shows specificity to stimulus timing and is particularly sensitive to unexpected sensory stimuli (Liu et al. 2008). More support for inferior olive involvement in timing came from an fMRI study conducted by Teki et al. (2011), where participants assessed the difference in duration of two successive time intervals when either presented with an irregular (absolute timing) or a regular (relative timing) sequence of clicks. They found that a network involving the inferior olive and the cerebellum operated as a precision clock to mediate absolute, duration-based timing (Teki et al. 2011). The inferior olive’s role in stimulus timing was further strengthened by an event-related fMRI study by Wu et al. (2011). Here, subjects had to indicate when they observed a change in the timing of presented single visual stimuli. Notably, the changes were quite close to the participant’s detection threshold, and consequently, in only about half of the trials, subjects were aware of such changes (Wu et al. 2011). The inferior olive demonstrated a robust response to time changes, independent of the subjects’ awareness (Wu et al. 2011).

Although the present investigation does not explicitly involve the perception or generation of timed intervals, our results are consistent with those reported by Liu et al. (2008), who suggested a particular sensitivity of the inferior olive to unexpected stimuli as the presented stimuli are familiar but ‘surprisingly’ do not match expectations. In our case, the familiar stimuli are repeated letter sequences instead of temporal sequences, and the surprising event is an unexpected probe letter. Electrophysiological studies conducted in animals render further support as they revealed activation of the inferior olive and climbing fibers to unexpected sensory events encountered during a familiar movement sequence (Gellman et al. 1985; Kim et al. 1987; Bloedel and Bracha 1998).

Thus, the results from this study underline that inferior olive activation is related to sequencing and not simply to visual stimulation given that this study did not use purely visual (encoding was auditory, and retrieval was visual from auditory) further highlighting that inferior olive activation is not only related to temporal properties of stimuli as previously reported (Xu et al. 2006; Liu et al. 2008; Teki et al. 2011) but is also relevant for encoding novel information and providing dynamic error feedback during retrieval. In contrast, the relatively lower level of activation observed during maintenance is consistent with a reduced role of inferior olive error processing functions occurring in the maintenance phase.

So far, in the context of learning and error feedback processing, inferior olive activation has only been investigated in a small number of studies and mostly in sequence timing tasks (Xu et al. 2006; Liu et al. 2008; Teki et al. 2011), limiting the comparability of results. In future verbal working memory studies, it would be of interest to analyze inferior olive activation in response to solely visually presented stimuli to investigate potential differences deriving from stimulus modality (auditory versus visual). It would also be of interest to investigate the involvement of the inferior olive in further working memory paradigms. Regardless of the type of working memory, the results of the present investigation suggest that it is crucial to analyze inferior olive activation separately for matching versus non-matching probe trials, as well as for novel versus repeating stimuli, in order to assess potential dynamic error signaling patterns.

## Data Availability

Data supporting the current study’s findings are available from the corresponding author upon reasonable request.
